# Overexpression of ZNT1 and NRAMP4 from the Ni Hyperaccumulator *Noccaea caerulescens* Population Monte Prinzera in *Arabidopsis thaliana* Perturbs Fe, Mn, and Ni Accumulation

**DOI:** 10.3390/ijms222111896

**Published:** 2021-11-02

**Authors:** Elisa Fasani, Giovanni DalCorso, Gianluca Zorzi, Caterina Agrimonti, Rosaria Fragni, Giovanna Visioli, Antonella Furini

**Affiliations:** 1 Department of Biotechnology, University of Verona, Str. Le Grazie 15, 37134 Verona, Italy; elisa.fasani@univr.it (E.F.); giovanni.dalcorso@univr.it (G.D.); gianluca.zorzi@univr.it (G.Z.); 2 Department of Chemistry, Life Sciences and Environmental Sustainability, University of Parma, Parco Area delle Scienze 11/a, 43124 Parma, Italy; caterina.agrimonti@unipr.it; 3 SSICA, Experimental Station for the Food Preserving Industry, Viale F. Tanara 31/A, 43121 Parma, Italy; rosaria.fragni@ssica.it

**Keywords:** heavy metals, hyperaccumulation, *Noccaea caerulescens* ecotype Monte Prinzera, heavy metal accumulation in plants

## Abstract

Metalliferous soils are characterized by a high content of metal compounds that can hamper plant growth. The pseudometallophyte *Noccaea caerulescens* is able to grow on metalliferous substrates by implementing both tolerance and accumulation of usually toxic metal ions. Expression of particular transmembrane transporter proteins (e.g., members of the ZIP and NRAMP families) leads to metal tolerance and accumulation, and its comparison between hyperaccumulator *N. caerulescens* with non-accumulator relatives *Arabidopsis thaliana* and *Thlaspi arvense* has deepened our knowledge on mechanisms adopted by plants to survive in metalliferous soils. In this work, two transporters, *ZNT1* and *NRAMP4,* expressed in a serpentinic population of *N. caerulescens* identified on the Monte Prinzera (Italy) are considered, and their expression has been induced in yeast and in *A. thaliana*. In the latter, single transgenic lines were crossed to test the effect of the combined over-expression of the two transporters. An enhanced iron and manganese translocation towards the shoot was induced by overexpression of *NcZNT1*. The combined overexpression of *NcZNT1* and *NcNRAMP4* did perturb the metal accumulation in plants.

## 1. Introduction

Plants adopt different strategies to survive in extreme environments, such as metalliferous soils. Some species have evolved tolerance mechanisms to cope with high concentrations of metal(oid)s. Most metal-tolerant species are excluders, able to reduce the uptake and translocation of trace metals; on the other hand, other species, defined hyperaccumulators, have evolved the ability to accumulate high amounts of trace elements in the aboveground tissues and to tolerate them without showing toxicity symptoms (for review see [1,2,3]). The increasing research focus on these species is justified by the interest for their evolutionary history and their peculiar adaptation to extreme metalliferous soils, that may help to understand the mechanisms controlling metal homeostasis in plants [3]. At the same time, they have a potential applicative value for the phytoremediation of heavy metal-polluted soils [4,5], as well as for the ambitious technology of phytomining, i.e., the commercial phytoextraction of highly valuable elements from metalliferous soils [6].

The hyperaccumulation trait was reported in a wide range of species belonging to distantly related families. Among hyperaccumulators, Brassicaceae and Phyllantaceae are the most highly represented [2]. The majority of them are nickel (Ni) hyperaccumulators, found in a large number of naturally Ni-enriched serpentine soils worldwide [7,8]. Among hyperaccumulators, pseudometallophyte *Noccaea caerulescens* (formerly *Thlaspi caerulescens*), belonging to the Brassicaceae family, has been used as a model for the study of hypertolerance and hyperaccumulation. This species shows remarkable intraspecific differences in its behavior toward metals: indeed, hypertolerance/accumulation of zinc (Zn) is constitutive but present at different degrees, whereas that of cadmium (Cd) is only characteristic of some calamine populations and that of Ni of serpentine ones [9,10].

The hypertolerance and hyperaccumulation traits are the result of the enhancement of physiological processes such as root metal uptake, symplast mobility, xylem loading and unloading, and is associated with a greater ability to counteract the toxicity of the metal by chelation and vacuolar sequestration [1,3]. As for metal chelation, for instance, nicotianamine seems to have a role in hyperaccumulation of Ni in *N. caerulescens* and of Zn and Cd in *Arabidopsis halleri* [11]. Also organic acids, such as oxalate, malate, and citrate, are reported to accumulate at a constitutively higher level in hyperaccumulators, as well as metallothioneins and phyochelatins [11]. On the side of molecular determinants, comprehensive analyses suggest that in hyperaccumulator species, behavioral variations are mainly associated with different gene copy number and expression regulation rather than acquisition of novel genes and functions [3]. Fundamental steps leading to metal tolerance and accumulation are carried out by transmembrane transporter proteins. Among them, members of the ZIP (Zinc-regulated transporter, Iron-regulated transporter Protein) and NRAMP (Natural Resistance-Associated Macrophage Protein) families showed differential regulation in *N. caerulescens* compared to non-accumulators *Arabidopsis thaliana* and *Thlaspi arvense* [12,13] and between populations with different edaphic behaviors [14]. In particular, *NcZNT1*, homologous to *A. thaliana ZIP4*, is expressed at higher levels in *N. caerulescens* in comparison to non-accumulators [12,15] and was proposed to have a role in Zn uptake in the roots [16] as well as in long distance transport [17]. Likewise, tonoplast transporter NRAMP4 has higher expression in *N. caerulescens* than in *A. thaliana* [18]. In addition, the transcription of both genes is induced upon moderate Ni treatment [19] and *NcNRAMP4* is expressed at higher levels in a Ni-hyperaccumulating population in comparison to calamine ones [14], suggesting that they may participate in the response to Ni, similarly to what was proposed in Ni-hyperaccumulator *Noccaea japonica* (formerly *Thlaspi japonicum*; [20]).

In biotechnological approaches, transgenesis helps the gene transfer between species, conferring new functions and abilities to plant species of particular interest. Metal accumulation in plants have been studied with the potential exploitation in two apparently opposite contexts. The first, to create plants to be applied in phytoremediation as reported above [5,6], characterized by enhanced abilities of translocation and accumulation of toxic metal ions into their above-ground tissues, removing them from the soils. The second, to enhance accumulation of useful metal ions in edible organs (e.g., iron), obtaining biofortified food. Considering these fields of applicability, in this work, the two metal transporters, ZNT1 (belonging to the ZIP family) and NRAMP4 from *N. caerulescens* ecotype Monte Prinzera (MP, Italy) were selected for further studies. The MP ecotype is a Ni hyperaccumulator growing on serpentinite, a soil rich in Ni, Co, and Cr; previous investigations demonstrated that this ecotype tolerates extremely high levels of Ni in soil, accumulates up to 12,000 mg kg^−1^ DW Ni in shoots and has high Ni translocation capacity [21]. In this work, *NcZNT1* and *NcNRAMP4* expression was tested in *N. caerulescens* MP upon different Ni treatments. The two genes were then expressed in yeast and *A. thaliana*, with the aim of analyzing the effect on metal accumulation induced by the expression of the two heterologous genes, alone and together. Therefore, single Arabidopsis transgenic lines were crossed to test the effect of the combined over-expression of *NcNRAMP4* and *NcZNT1*.

## 2. Results

### 2.1. The Expression of NRAMP4 and ZNT1 in Ni hyperaccumulator N. caerulescens MP

**Figure 1:**
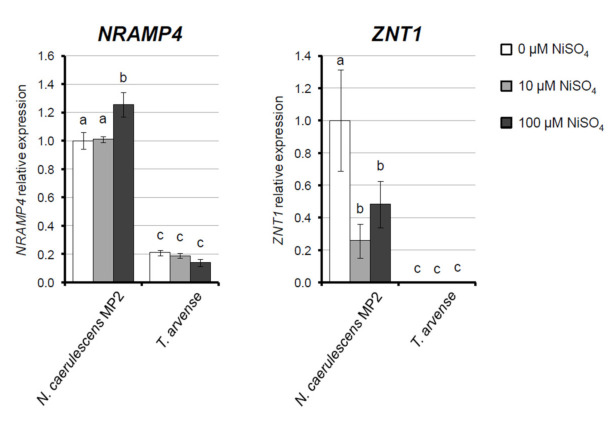
Expression profiles of *NRAMP4* and *ZNT1* tested in leaves of the Ni hyperaccumulator *Noccaea caerulescens* MP (population MP2) and of the non-accumulator *Thlaspi arvense,* both grown in hydroponic culture in the absence (white bars) or presence of 10 μM (grey bars) and 100 μM (black bars) NiSO_4_ for 24 h. The expression level of *NRAMP4* and *ZNT1* is expressed as relative expression on internal reference genes actin 2, 8, and ubiquitin 10 according to the Livak—Schmittgen 2^−ΔΔCT^ method. Statistically significant variations, as resulting from the ANOVA test (*n* = 3) followed by a post hoc Tukey’s test, at *p* < 0.05, are marked by different letters. Bars correspond to standard error.

The *N. caerulescens* MP population, MP2, that has been studied in this work grows on a site that is poorly vegetated and characterized by the presence of serpentine rock, with low organic matter content and high Ni and Fe levels [21,22]. In situ, MP2 population is characterized by a very high root-to-shoot translocation factor for Ni and accumulates about 6000 mg kg^−1^ Ni in the shoot [21]; this population has been also reported to accumulate substantial levels of Fe and Mn [23]. The expression of *NRAMP* and *ZNT1* was tested in leaves of *N. caerulescens* MP grown in hydroponic culture in the absence or in the presence of 10 μM and 100 μM NiSO_4_ for 24 h, and compared with that of the non-accumulator *T. arvense* (Figure 1). In *N. caerulescens* MP, both genes are expressed at significantly higher levels than in *T. arvense* in all growth conditions. In particular, *NcZNT1* expression was present in all conditions tested but down-regulated after 24 h of NiSO_4_ treatment independently of the concentrations, whereas in *TaZNT1* was undetectable either in the absence or the presence of Ni. On the other hand, *NcNRAMP4* showed constitutively high expression in *N. caerulescens* MP, increasing upon 24 h of 100 μM NiSO_4_ treatment; overall, its transcription was approximately five-fold higher than the orthologous gene in *T. arvense* (Figure 1).

### 2.2. Expression of ZNT1/ZIP4 and NRAMP4 from N. caerulescens and A. thaliana Has Contrasting Effects in Yeast

**Figure 2:**
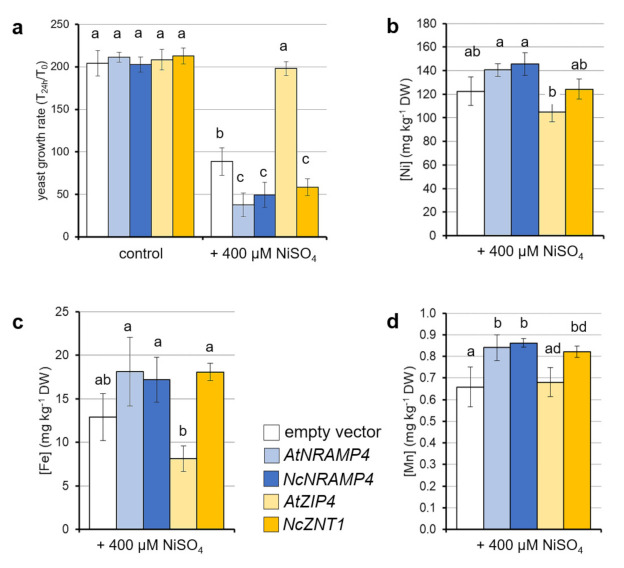
Effect of *NcNRAMP4, AtNRAMP4, NcZNT1,* and *AtZIP4* heterologous expression in yeast cells. (**a**) Yeast strains transformed with the empty pADSL vector (white bars) and with the recombinant vectors (see legend for the colour code) were grown in liquid YNB/G/W- medium (control) or YNB/G/W- supplemented with 400 µM NiSO_4_. The cultures were grown at 28 °C under constant agitation at 160 rpm for 24 h and OD was measured at the end. The growth rate was expressed as OD_24hr_/OD_T0_. The analysis was performed in triplicate and repeated in three independent experiments; data presented refer to a single trial representative of the three replicates. Statistically significant variations, as resulting from the ANOVA test (*n* = 4), are marked by different letters. Bars correspond to standard deviation. Metal accumulation—(**b**) Ni; (**c**) Fe; and (**d**) Mn—was determined on 24 h-grown cultures as described in M&M. Statistically significant variations at *p* < 0.05, as resulting from the two-way ANOVA test (*n* = 3), followed by a post-hoc Bonferroni test, are marked by different letters. Bars correspond to standard deviation.

Yeast lines expressing *ZNT1/ZIP4* and *NRAMP4* from *N. caerulescens* and *A. thaliana* were tested for Ni tolerance and metal accumulation, in comparison to yeast transformed with the empty pADSL vector as a control. When yeast was grown in the absence of NiSO_4_ for 24 h, no differences were found among the different genotypes in terms of growth rate (Figure 2a). In the presence of 400 µM NiSO_4_, the yeast line expressing *AtZIP4* showed a significant increase in the growth rate in comparison to all other genotypes; on the contrary, its ortholog *NcZNT1* significantly decreased the growth rate in the 24 h in comparison to the empty vector control. Analogously, *AtNRAMP4 and NcNRAMP4* expression in yeast led to a significant decrease in growth rate upon Ni treatment, in comparison to the yeast transformed with the empty pADSL vector (Figure 2a).

As for metal accumulation, contents of Ni, Fe, and Mn were assessed in the yeast lines grown for 24 h in the presence of 400 µM NiSO_4_. Fe and Mn were chosen since they share interactions with Ni in plant mineral nutrition [24]. Overall, Ni concentration in the different yeast transformants correlated negatively with the growth rate. In particular, the *AtZIP4*-expressing line, more Ni-tolerant, and the *AtNRAMP4/NcNRAMP4*-expressing lines, Ni-sensitive, accumulate less and more Ni than the empty vector control, respectively (Figure 2b). Analogously, profiles for the other metals tested were similar to that of Ni (Figure 2c,d). In particular, both Fe and Mn concentrations were significantly higher in *NcZTN1-* and *NRAMP4*-expressing lines with respect to the control; *AtZIP4* only differed from the empty vector line for the lower Fe content (Figure 2c,d).

### 2.3. Expression of NcNRAMP4 and NcZNT1 in A. thaliana Alters Plant Growth and Metal Accumulation

**Figure 3:**
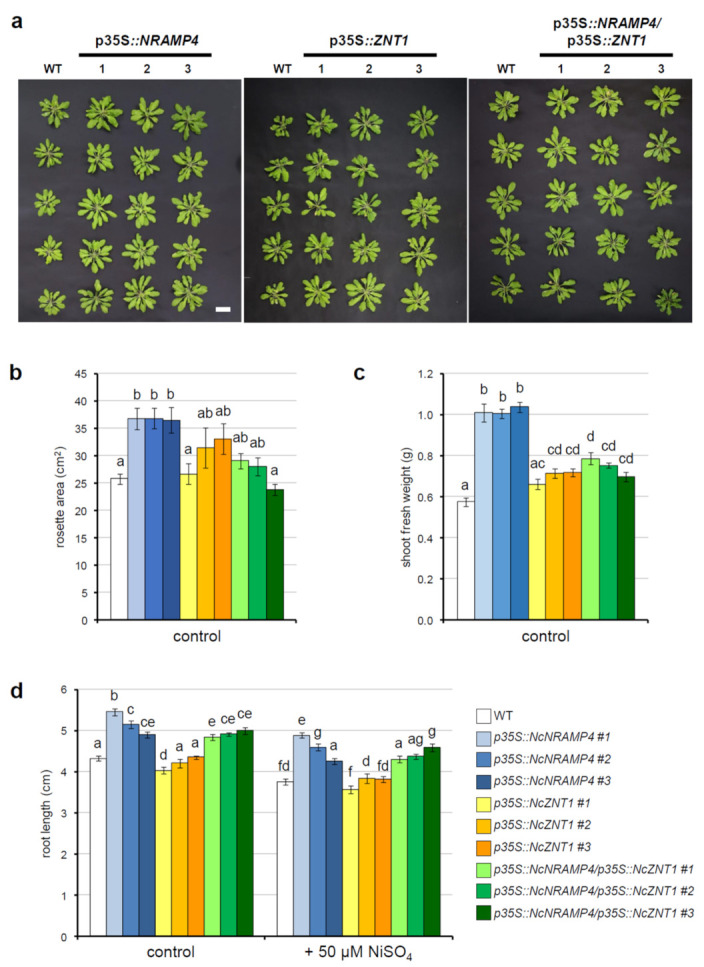
Effect of the expression of *NcNRAMP4* and *NcZNT1* in *Arabidopsis thaliana* Col_0 plants. (**a**) Picture of the transgenic and WT plants grown in soil under control conditions in a greenhouse under a 16 h light/8 h dark regime at 22 °C/18 °C (light intensity of 80 to 120 μmol m^−2^ s^−1^). (**b**) Rosette area and (**c**) biomass were measured on four-week-old plants. (**d**) Primary root length of plants of *Arabidopsis thaliana* wild-type and transformed lines with *NcNRAMP4* and *NcZNT1* alone or in combination grown for 10 days, in vitro in Gelrite-solidified MS medium, either standard (control) or supplemented with 50 μM NiSO_4_ (Ni excess) as described in M&M. Statistical analyses were performed by two-way ANOVA (*n* = 18) followed by a post-hoc Bonferroni test. Statistically significant variations, at *p* < 0.05, are marked by different letters.

To investigate the possible role of *NcZNT1* and *NcNRAMP4* in plant metal homeostasis associated with serpentine soils, both genes were over-expressed in *A. thaliana*, either singly (p35S*::NcNRAMP4* and p35S*::NcZNT1* lines) or in combination (p35S*::NcNRAMP4/*p35S*::NcZNT1*). Three independent lines, displaying the highest expression levels for the transgenes, were selected for each transformed genotype and compared with wild-type *A. thaliana*. When grown in soil for four weeks, p35S*::NcNRAMP4* lines had consistently wider rosette area and fresh biomass than wild-type plants; on the other hand, no significant difference in growth was visible in p35S*::NcZNT1* plants in comparison to wild-type, despite two lines having slightly higher fresh weight (Figure 3a–c). Double-transformed lines were intermediate in size: although differences in rosette area were not significant, transgenic plants had higher shoot fresh weight (Figure 3a–c).

Consistently, when grown in vitro in standard MS medium for 17 days, p35S*::NcNRAMP4* and double transformed p35S*::NcNRAMP4/*p35S*::NcZNT1* lines had longer primary roots, whereas p35S*::NcZNT1* plantlets did not show differences with wild-type (Figure 3d). To evaluate plant tolerance to Ni excess, one-week-old plantlets were transferred to MS medium supplemented with 50 μM NiSO_4_ for 10 days. The applied Ni treatment had a moderate negative effect on root length, and differences between the genotypes were maintained also under Ni excess (Figure 3d).

**Figure 4:**
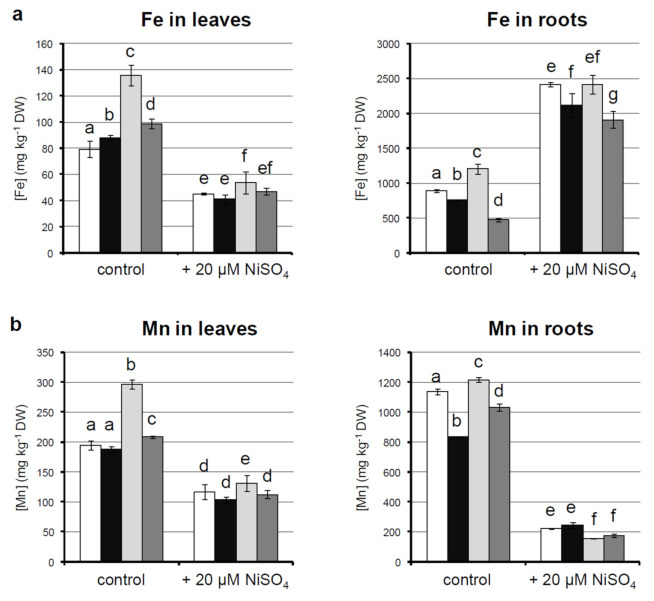

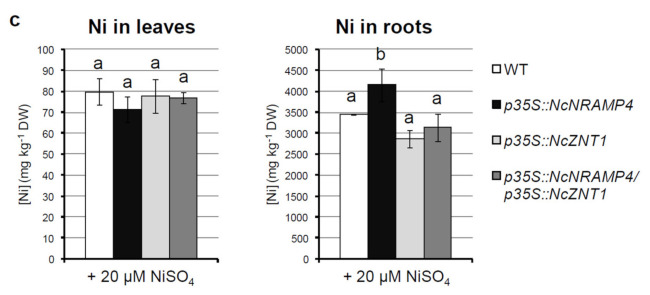
Metal accumulation in *Arabidopsis thaliana* Col_0 plants overexpressing *NcNRAMP4* and *NcZNT1.* For metal quantification in roots and shoots—(**a**) Fe, (**b**) Mn, and (**c**) Ni—plants were grown in hydroponic solution, as described in M&M, under control conditions or treated with 20 µM NiSO_4_ for two weeks. Leaves and roots for each line were collected separately in triplicate, ground to fine powder in liquid nitrogen and concentrations of Ni, Fe, and Mn were determined by ICP-AES. Statistical analyses were performed by two-way ANOVA (*n* = 6) followed by a post-hoc Bonferroni test. Statistically significant variations, at *p* < 0.05, are marked by different letters.

To elucidate the reason behind the observed differences in growth of p35S*::NcNRAMP4* and p35S*::NcNRAMP4/*p35S*::NcZNT1* plants, metal homeostasis was further investigated. For this purpose, the accumulation and distribution of Ni and the associated metals Fe and Mn was tested on plants grown for two weeks in hydroponic culture in Hoagland’s solution alone (control) or supplemented with 20 μM NiSO_4_. Plants from all lines tested showed a similar phenotype to that observed in soil and in vitro, and symptoms of toxicity due to excess Ni were not observed (data not shown). Upon control conditions, p35S*::NcZNT1* plants had significantly higher contents of Fe and Mn in both shoots and roots than all other genotypes (Figure 4a,b). On the contrary, p35S*::NcNRAMP4* and double transformed plants accumulated less Fe and Mn in roots than both control and *ZNT1*-expressing plants; Fe concentration in leaves was higher, whereas Mn was slightly higher in p35S*::NcNRAMP4/*p35S*::NcZNT1* plants kept in control conditions and did not show statistically significant differences in p35S*::NcNRAMP4* ones when compared to wild-type (Figure 4a,b). Upon Ni treatment, Fe content decreased in leaves and increased in roots in all genotypes considered; Fe concentration in leaves was higher for p35S*::NcZNT1* plants than wild-type and the other transgenic lines, whereas p35S*::NcNRAMP4* and double transformed plants had less Fe in roots (Figure 4a). Mn concentration decreased upon Ni treatment in both roots and shoots of all genotypes tested (Figure 4b). Ni content was also measured in plants maintained in the presence of Ni. No effect was observed on Ni accumulation in the shoot for the over-expression of the single transporters or their combination, whereas a significant increase of root Ni concentration was measured in p35S*::NcNRAMP4* plants (Figure 4c).

Translocation factors (TFs), calculated as the ratio between metal concentration in shoots and metal concentration in roots, confirm that Fe translocation is impaired by Ni treatment; on the other hand, Mn TFs increased upon high Ni (Table 1). Fe TF is higher in double transformed plants, although such difference is absent when plants were grown in the presence of Ni. As for Mn, p35S*::NcZNT1* plants had higher TFs upon both growth conditions, whereas Mn TF for p35S*::NcZNT1* plants was higher only in control conditions (Table 1).

## 3. Discussion

Due to their peculiar strategies to deal with extreme environments characterized by imbalanced mineral compositions, metallophytes and in particular metal hyperaccumulators represent a valuable tool for the study of metal homeostasis as well as adaptation to heavy metal-enriched soils. However, although Ni hyperaccumulators constitute a vast majority of all known hyperaccumulators [7,8], the understanding of mechanisms ensuring adaptation to serpentine soils is still somewhat poor [25].

In this work, two metal transporters, NcZNT1 and NcNRAMP4, from the Ni hyperaccumulator *N. caerulescens* population Monte Prinzera (MP), were selected for further analysis. Previous evidences suggest that these transporters may play roles in the adaptation to serpentine soils, since their orthologues in *N. japonica* have been demonstrated to alter Ni tolerance and accumulation in yeast [20]. Interestingly, both genes were up-regulated in *N. caerulescens* MP in the presence of 10 μM Ni as opposed to Ni absence, after 28 days of treatment [19]. For the short term (one day), we evaluated the expression of *ZNT1* and *NRAMP4* by comparing *N. caerulescens* MP with non-tolerant, non-accumulator *T. arvense*. The higher constitutive expression observed in *N. caerulescens* MP is coherent with the expression profiles of metal homeostasis genes in hyperaccumulator species. In particular, both *ZNT1* and *NRAMP4* were expressed at higher levels in various *N. caerulescens* populations than in related non-accumulators *A. thaliana* and *T. arvense* [15,16,18].

Keeping in mind that heterologous expression in yeast may not reflect the transport activity that the transporters show in the plant cell], when *NcNRAMP4* and *NcZNT1* genes were expressed in yeast, as well as their *A. thaliana* orthologues as a control, they altered both Ni tolerance and metal accumulation. In particular, marked differences in Ni tolerance were observed between genotypes, in contrast with only moderate variations in Ni accumulation. Similarly to their *A. thaliana* orthologues, *NRAMP4* and *ZNT1* from Ni hyperaccumulator *N. japonica* respectively reduced and increased Ni tolerance in transformed yeast, the former by raising cellular Ni content [20]. Interestingly, also in that case, differences in Ni accumulation were either low or absent, particularly for *ZNT1*, and were explained by the authors as possibly due to low affinities or speed of the transporters [20]. On the other hand, it is well known that Fe and Mn impact on Ni tolerance and accumulation in both plants and yeast [26,27,28], and indeed the contents of both metals vary significantly in transformed yeasts in this work. This result suggests that the observed differences in Ni tolerance are the result of an altered global metal homeostasis rather than specific transporter activity toward Ni.

Interestingly, the phenotype of yeast strains transformed with the *N. caerulescens ZNT1* is different than that with the *A. thaliana* orthologue, while there is no difference in behavior of yeast harboring *AtNRAMP4* or *NcNRAMP4*. Considering the aminoacidic sequences of the two proteins, while AtNRAMP4 and NcNRAMP4 are basically the same protein, sharing 93% identity and 97%similarity, AtZIP4 and NcZNT1 differ, sharing 89% identity and 93% similarity, with the main differences in the putative in the region involved in metal binding (data not shown). These differences can be the reason for a diverse metal binding specificity. Although no further evidence is present regarding these genes and their involvement in the response to Ni, previous researches indicate that both *AtNRAMP4* and *NcNRAMP4* (from the Zn-hyperaccumulating population La Calamine) were able to complement yeast mutant strains lacking Fe and Mn uptake [18,29,30]. This is consistent with the higher Fe and Mn contents in Ni-treated *AtNRAMP4-* or*NcNRAMP4-*expressing yeast line here observed, and points to a functional equivalence between AtNRAMP4 and NcNRAMP4 as previously postulated [18]. *NcZNT1* was proposed as specific for Zn, with no activity in transport of Cu, Fe, or Mn, while Cd was transported at a low affinity into yeast cells [17]. Conversely, *AtZIP4* was confirmed to transport Zn and Cu, although other metals were not tested. In this work, *AtZIP4* expressing strain had the highest Ni tolerance, as associated with lower levels of cellular Ni and especially Fe, suggesting that at least in yeast and in contraposition with NcZNT1, this transporter acts in removing these metal from the cytosol.

To investigate whether NcZNT1 and NcNRAMP4, constitutively expressed in *N. caerulescens* ecotype MP, might play a role in Ni accumulation and tolerance or affect the transport of other metal ions in the presence of Ni, both genes were over-expressed in *A. thaliana. NcNRAMP4* overexpression enhanced plant growth, in term of both shoot and root growth. Interestingly, such effect of NcNRAMP4 seems to be independent of the accumulation of Fe or Mn, since none of them accumulate to higher extent in transgenic plants. Similarly, AtNRAMP4 was showed not to influence translocation and accumulation of metals in shoots also by [31]. On the contrary, NcNRAMP4 constitutive expression in *N. caerulescens* MP might be important to enhance cycling of metal ions from vacuoles of root cells, a prerequisite for their prompt root-to-shoot transport.

A different situation is encountered in transgenic plants overexpressing *NcZNT1*. This protein plays an important role in *N. caerulescens*, being involved in establishing metal influx (mainly Zn and Cd) into the root vasculature, the first step of the root-to-shoot transport sustaining metal hyperaccumulation in shoots [32]. As already reported for *A. thaliana* overexpressing *NcZNT1* of the accession La Calamine, Belgium [32], also MP—NcZNT1 enhanced Mn accumulation only in transgenic *A. thaliana* shoots, and Fe in both shoots and roots, and this could be due to a perturbed Zn homeostasis and distribution with consequent modulation of expression of genes encoding transporter proteins [32].

When both transporters were expressed, the dramatic effect of NcZNT1 on Fe and Mn shoot translocation was weakened, but the translocation factor for Fe was enhanced in transgenic plants, pointing to a cooperative effect of the expression of both proteins in increasing Fe translocation towards the shoot.

The effects were harshly diminished upon treatment with Ni, which is greatly accumulated into the root of both wild-type and transgenic plants, with a slight overaccumulation of Ni in roots of plants overexpressing NcNRAMP4. Excess Ni interferes with homeostasis of several metals, mainly Fe. The latter is required in larger amounts respect to others for its role as a cofactor in proteins participating in photosynthesis and respiration as well as in antioxidant activity [33]. Indeed, in the Ni hyperaccumulator *Alyssum inflatum,* toxicity symptoms due to excess Ni are mainly caused by a worsening of Fe-dependent protein functions in shoots due to disruption of root-to-shoot Fe translocation [34]. In this species, an increased Fe uptake was enhanced when plants were exposed to high Ni concentrations [34]. Excess Ni decreased the activity of antioxidant Fe enzymes causing oxidative stress associated with the competition of Ni with Fe [35]. In *A. thaliana* excess Ni induces Fe deficiency and a subsequent increased expression of *IRT1* [36]. Also in *N. caerulescens* MP, the treatment with Ni increased Fe accumulation and retention in roots, and indeed also Fe-associated TF greatly diminished. In this case, all transgenic genotypes behaved in a similar manner. Interestingly, even though Mn accumulation was reduced by Ni treatment in both shoots and roots, its TF increased, again pointing to a general effect of Ni in perturbing metal distribution through the plant body. Also in this case, Ni effect was independent from the transgene expression in plants, except for plants overexpressing NcNRAMP4, which showed higher Ni retention in roots at the expense of its shoot accumulation. Therefore, NcNRAMP4 does not cooperate to Ni transport and accumulation to the shoot. On the other hand, since the biological needs for Ni are usually very low, several studies speculated that Ni-transport in plant cells may be carried out in combination with transport of other metals, such as those deputed to Zn or Fe transport [20]. Indeed, when expressed in yeast, NRAMP4 identified in the Ni hyperaccumulator *N. japonica* caused increased Ni sensitivity and accumulation, indicating its role in Ni homeostasis [20].

In conclusion, the results of this work indicate that NcNRAMP4 and NcZNT1, although highly expressed in *N. caerulescens* MP, are probably not the main actors involved in Ni transport to the shoot. Considering the expression in yeast, the differences between the phenotype of yeast strains transformed with the *N. caerulescens ZNT1* and with the *A. thaliana* orthologue could be due to the variation in their sequences and further research in this context would highlight important domains for metal ion specificity. The overexpression of these genes, separately or together, altered the metal homeostasis in plants by interfering with the transport of Fe and Mn. Hence, in programming biotechnological works to tailor the transport and accumulation of essential metal(oid)s in edible plant parts (i.e., biofortification of food crops) or to increase the amount of toxic elements in epigeous tissues of the plant body, for phytoremediation purposes, the changes caused in metal network have to be taken into account, and transfer of a single gene (in the case here reported, NcZNT1) is sometimes more effective than transferring more than one.

## 4. Materials and Methods

### 4.1. Plant Material and Growth Conditions

Seeds of *N. caerulescens* (J.Presl & C.Presl) F.K.Mey. ecotype Monte Prinzera were collected at the MP2 site (44.65096° N–10.08369° E), in the Northern Apennines (Italy). Non-accumulator *Thlaspi arvense* L., used in the comparison, was kindly provided by Dr. Claudio Varotto (Edmund Mach Foundation, San Michele all’Adige, Trento, Italy). Seeds were sterilized for 1 min with 70% ethanol and for 15 min with 20% sodium hypochlorite and 0.03% Triton X-100, then rinsed three times with sterile water. Sterilized seeds were sown in vitro on solid MS medium (Merck KGaA, Darmstadt, Germany) [37] supplemented with 10 g L^−1^ sucrose; germinated plants were maintained for three weeks in a growth chamber under a 16 h light/8 h dark regime at 22 °C/18 °C (light intensity of 80 to 120 μmol m^−2^ s^−1^). For in vivo experiments, *N. caerulescens* and *T. arvense* plants were transferred to hydroponic culture in Hoagland’s solution [38]. After two weeks of acclimation, plants were transferred in Hoagland’s solution either in the absence of Ni or supplemented with 10 µM or 100 µM NiSO_4_ for one day. Shoots of both plant species were collected to perform gene expression analysis; three pools of two plants each were sampled for each species and condition as biological replicates.

### 4.2. ZNT1 and NRAMP4 Expression Analysis in N. caerulescens and T. arvense

Total RNA was extracted with TRIzol Reagent (Thermo Fisher Scientific, Waltham, MA, USA). After DNase treatment, first-strand cDNA was synthesized using the Superscript III Reverse Transcriptase Kit (Thermo Fisher Scientific, Waltham, MA, USA). Gene expression analysis was assessed by real-time reverse transcription polymerase chain reaction (RT-PCR), using the StepOnePlus Real-Time PCR System (Thermo Fisher Scientific, Waltham, MA, USA) and the KAPA SYBR FAST ABI Prism 2X qPCR Master Mix (Kapa Biosystems, Wilmington, MA, USA). Primers for the analysis of *ZNT1* and *NRAMP4* were designed to amplify orthologous genes in the Brassicaceae family and are reported in Table 2; actin 2 and 8 (At3g18780/At1g49240) and ubiquitin 10 (At4g05320) were used as internal reference genes for sample normalization. Amplification data were analyzed by the 2*^−^*^ΔΔCT^ method [39].

### 4.3. Isolation of NcNRAMP4, NcZNT1, AtNRAMP4 and AtZIP4 Coding Sequences, Plasmid DNA Constructs Preparation and Yeast Transformation

Complete coding sequences of *NcNRAMP4, NcZNT1*, *AtNRAMP4,* and *AtZIP4* were amplified from *N. caerulescens* MP2 and *A. thaliana* Columbia (Col-0) cDNAs, respectively, using the primers reported in Table 2 and the Platinum Pfx DNA polymerase (Thermo Fisher Scientific, Waltham, MA, USA), according to the manufacturer’s instructions. Sequence information of NcNRAMP4 and NcZNT1 has been loaded onto the NCBI database, with codes OK322355 and OK322354, respectively. All sequences were cloned in the pGEM-t easy vector (Promega, Madison, WI, USA) for sequencing, then excised using the *BamH*I and *Eco*RI (*Xho*I for *AtZIP4*) restriction enzymes and ligated in the pADSL expression vector downstream the ADH promoter (DualSystem Biotech, Schlieren, Switzerland). *Saccharomyces cerevisiae* strain DY1457 (*MATɑ, his3, leu2, trp1, ura3-52*) was transformed with the empty pADSL vector as a control and with pADSL vector harboring *NcNRAMP4*, *NcZNT1*, *AtNRAMP4,* and *AtZIP4*, using the lithium acetate/single-stranded carrier DNA/polyethylene glycol method [40]. The selection of transformants was performed on YNB/G/W- medium, containing 0.7% yeast nitrogen base (YNB; Sigma-Aldrich, St. Louis, MO, USA), 1.92 g/L of each amino acid except Trp (Merck KGaA, Darmstadt, Germany) and 2% glucose.

### 4.4. Analysis of Ni Tolerance and Accumulation in Yeast

To define the Ni concentration for tolerance and accumulation assays, minimum inhibitory concentration (MIC) was determined in yeast cells transformed with the empty pADSL vector by spot assay. Yeast was grown overnight in 5 mL selective liquid YNB/G/W- at 28 °C to early stationary phase. Yeast cells were then diluted to OD_600_ nm = 1, 0.1, 0.01 and 0.001 and spotted on YNB/G/W- plates supplemented with 0, 200, 400, 600, 800 µM NiSO_4_. The concentration of 400 µM NiSO_4_ reduced the growth of yeast transformed with empty plasmid at 1 and 0.1 OD, and inhibited the growth of yeast at 0.01 and 0.001 OD, whereas higher concentrations completely abolished yeast growth (Supplementary Figure S1). This concentration was therefore identified as MIC and was chosen for growth experiments in liquid culture.

Yeast strains transformed with the empty pADSL vector and with the recombinant vectors were grown in liquid YNB/G/W- medium for 24 hrs; they were then inoculated to OD_T0_ = 0.05 into 20 mL liquid YNB/G/W- (control) or YNB/G/W- supplemented with 400 µM NiSO_4_. The cultures were grown at 28 °C under constant agitation at 160 rpm for 24 h and OD measured at the end. The growth rate was expressed as OD_24hr_/OD_T0_. The analysis was performed in triplicate and repeated in three independent experiments.

24 h-grown cultures were centrifuged at 10,000× *g* for 3 min; pellets were collected and washed three times with ultrapure water to determine metal concentration in yeast.

### 4.5. Plasmid DNA Constructs Preparation and A. thaliana Transformation

Complete coding sequences of *NcNRAMP4* and *NcZNT1* were amplified from *N. caerulescens* MP2 cDNA using the primers reported in Table 2 and the Platinum Pfx DNA polymerase (Thermo Fisher Scientific, Waltham, MA, USA), according to the manufacturer’s instructions. *NcNRAMP4* was cloned in the pDONR201 vector using the Gateway technology by BP recombination (Thermo Fisher Scientific), and then transferred to the expression vector pH2GW7, carrying the *hpt* gene as plant selectable marker, by LR recombination (Thermo Fisher Scientific, Waltham, MA, USA). *NcZNT1* was cloned in the pGEM-t easy vector (Promega, Madison, WI, USA); the coding sequence was then excised with the *BamH*I and *Xho*I restriction enzymes and ligated in the final expression vector pMD1, containing the *nptII* gene as plant selectable marker [41]. The pH2GW7-p35SCaMV*::NcNRAMP4* and pMD1-p35SCaMV*::NcZNT1* recombinant plasmids were introduced into *Agrobacterium tumefaciens* strains GV3101 and EHA105, respectively. Both strains were used to transform *A. thaliana* Col-0 by floral dip [42]. Transformed plants were selected on MS medium supplemented with hygromycin or kanamycin, according to the selective marker, and the presence of the transgene was verified by PCR on the genomic DNA. Expression of *NcNRAMP4* and *NcZNT1* was quantified by real-time RT-PCR as previously described. Homozygous T3 plants of the three highest expressing lines for each transformation were selected for further analysis.

Double over-expressing lines were obtained by crossing the highest *NcZNT1*-expressing line with the highest *NcNRAMP4*-expressing line, followed by selection on MS medium containing both kanamycin and hygromycin. PCR were performed on genomic DNA of plants derived from crossing and selected on medium supplemented with kanamycin and hygromycin to confirm the presence of both transgenes. Three independent transgenic lines for each transgene and three lines carrying both transgenes were chosen, propagated, and subjected to detailed analyses.

### 4.6. Analysis of A. thaliana Transgenic Lines

*A. thaliana* lines expressing *NcZNT1* and *NcNRAMP4* alone or in combination, as well as wild-type, were analysed for phenotype in soil under greenhouse conditions. Rosette area and biomass were measured on four-week-old plants.

Ni tolerance was tested in vitro on *A. thaliana* wild-type and transformed lines with *NcNRAMP4* and *NcZNT1* alone or in combination. Seeds were sterilized and germinated on MS medium as described above for one week. Eighteen plantlets for each line were then transferred to Gelrite-solidified MS medium, either standard (control) or supplemented with 50 μM NiSO_4_ (Ni excess), and grown vertically. Primary root length and shoot fresh biomass were measured after 10 days.

For metal quantification in roots and shoots, six two-week-old plantlets of each genotype, grown in vitro, were transferred in hydroponic culture in Hoagland’s solution and acclimated for one further week. Plants were then either kept in control conditions or treated with 20 µM NiSO_4_ for two weeks. Leaves and roots for each line were collected separately in triplicate, ground to powder in liquid nitrogen, and stored at −80 °C until utilization for metal quantification.

### 4.7. Metal Quantification in Yeast and A. thaliana Transgenic Lines

Samples of yeast and *A. thaliana* transgenic lines collected in the accumulation experiments were oven-dried at 60 °C and subjected to microwave-assisted acid digestion (EPA 3051A 2007). Concentrations of Ni, Fe, and Mn were determined by inductively coupled plasma atomic emission spectrometry (ICP-OES; EPA 6010C 2007).

Leaves, roots and yeast pellets were mineralized in duplicate by a high-pressure microwave-assisted acid digestion (1500 W, 150 Bar, 250 °C, 30 min, UltraWAVE Milestone, Sorisole, Italy), weighing in quartz tubes approximately 0.1 g of leaves and yeast pellets, 0.01 g of roots and adding 2 mL of ultrapure HNO_3_ (67–69% m/v, Chem-Lab NV, Pico-Pure Plus, Zedelgem, Belgium) for yeast pellets and ultrapure inverted aqua regia (HNO_3_:HCl:H_2_O, 3:1:1 *v/v/v*) for leaves and roots.

The digested solutions, 100 or 1000-fold diluted with high-purity deionized water (0.05 μS cm^−1^, Purelab^®^ Ultra ELGA, High Wycombe, UK) and filtered on 0.45 μm filters (Millex^®^-HA, Millipore), were introduced into ICP-OES spectrometer (Vista-MPX, Varian, Agilent Technologies, Santa Clara, CA) for Fe, Mn, Ni, and Zn quantification. The instrument configuration and measurement conditions were previously described [43].

Fe (259.940 nm), Mn (257.610 nm), Ni (231.604 nm), and Zn (213.857 nm) were quantified through calibration lines (0.001–1.6 mg/L for Mn, 0.010–3.0 mg/L for Fe and Zn and 0.1–50 mg/L for Ni), prepared from a customized multi-element solutions of Fe, Mn, and Zn (10 mg/L of Mn and 100 mg/L of Fe and Zn, TraceCERT^®^ Fluka Analytical, Sigma-Aldrich, St. Louis, MI, USA) and a 1000 mg/L mono-element standard solution of Ni (TraceCERT^®^ Fluka Analytical, Sigma-Aldrich, St. Louis, MI, USA).

### 4.8. Statistical Analysis

Data in histograms are represented as mean ± standard deviation or standard error as indicated in figure legends. Statistical significance of the data was evaluated using the GraphPad Prism 7 (GraphPad Software, San Diego, CA, USA). Statistical analyses were performed by one-way analysis of variance (ANOVA) followed by a post hoc Tukey’s test when a single variable (i.e., genotype) was associated, by two-way ANOVA followed by a post hoc Bonferroni test when two variables (i.e., genotype and treatment) were present. Statistically significant variations, at *p* < 0.05, are marked by different letters.

## Figures and Tables

**Table 1:** Translocation factors (TFs) for Fe, Mn, and Ni in wild-type (WT) and transformed plants grown in hydroponic culture in control conditions or upon Ni supplementation (+20 µM NiSO_4_). TFs are expressed as mean ± standard deviation; letters, when different, indicate statistical significance as evaluated by one-way ANOVA plus post-hoc Tukey’s test (*p* < 0.05).

	Control			+20 µM NiSO_4_		
Genotype	Fe	Mn	Ni	Fe	Mn	Ni
WT	0.09 ± 0.01 a	0.17 ± 0.01 a	-	0.02 ± 0.00 a	0.53 ± 0.07 ab	0.02 ± 0.00 a
p35S::*NRAMP4*	0.12 ± 0.00 a	0.23 ± 0.01 ab	-	0.02 ± 0.00 a	0.42 ± 0.07 a	0.02 ± 0.00 a
p35S::*ZNT1*	0.11 ± 0.00 a	0.24 ± 0.00 b	-	0.02 ± 0.00 a	0.85 ± 0.09 b	0.03 ± 0.00 a
p35S::*NRAMP4*/p35S::*ZNT1*	0.21 ± 0.01 b	0.20 ± 0.01 a	-	0.02 ± 0.00 a	0.65 ± 0.11 ab	0.02 ± 0.00 a

**Table 2:** Primers utilized to amplify NRAMP4 and ZNT1—ZIP4. See Material and Methods for details.

A. thaliana transformation		
*NcNRAMP4*	#F	GGGGACAAGTTTGTACAAAAAGCAGGCTATGTCGGAGACGGAGAGAGA
	#R	GGGGACCACTTTGTACAAGAAAGCTGGGTCTAATTGCAAGGAGTGTACGT
*NcZNT1*	#F	*GGATCC*ATGATCATCGCCGATCTTCTTTG
	#R	*CTCGAG*CTAAGCCCAAATGGCGAGTG
**Yeast complementation**		
*NcNRAMP4*	#F	*GGATCC*ATGTCGGGAGACTGATAGAGAG
	#R	*GAATTC*CTAATTGCAAGGAGTGTACGT
*NcZNT1*	#F	*GGATCC*ATGATCATCGCCGATCTTCTT
	#R	*GAATTC*CTAAGCCCCAAATGGCGAGTG
*AtNRMAP4*	#F	*GGATCC*ATGTCGGAGACTGATAGAGAGCG
	#R	*GAATTC*CTCACTCATCATCCCTCTGTGG
*AtZIP4*	#F	*GGATCC*ATGGCTTCTTCTACCACTAAA
	#R	*CTCGAG*CTAAGCCCAAATGGCGAGAGCA
**Expression analysis by Real Time—RT PCR**		
*Nc-Ta NRAMP4*	#F_N4RT	TTCCCGATACTCTACATATGG
	#R_N4RT	CCATCGCATGTACCATGAGC
*Nc-Ta ZNT1*	#F_ZNTRT	TCAACTCGCATAGCCCTGG
	#R_ZNTRT	AGCCTCACATTACAACTCATC
*ACT2/8*	#F_ACT	AACATTGTGCTCAGTGGTGG
	#R_ACT	GACCTTAATCTTCATGCTGCT
*Ubi10*	#F_UBI10	GGACAAGGAAGGTATTCCTC
	#R_UBI10	CTCCTTCTGGATGTTGTAGTC

## Data Availability

All data supporting the findings of this study are available within the paper and within its Supplementary Materials published online.

## Supplementary Materials

The following are available online at https://www.mdpi.com/article/10.3390/ijms222111896/s1.
