# Supplementary material for: Overexpression of ZNT1 and NRAMP4 from the Ni Hyperaccumulator Noccaea caerulescens Population Monte Prinzera in Arabidopsis thaliana Perturbs Fe, Mn, and Ni Accumulation
Source: Int J Mol Sci. 2021 Nov 2;22(21):11896. doi: 10.3390/ijms222111896 (PMC8584810; doi:10.3390/ijms222111896)
Supplement: Supplementary file 1 [file ijms-22-11896-s001.zip › ijms-1429985-supplementary.pdf]

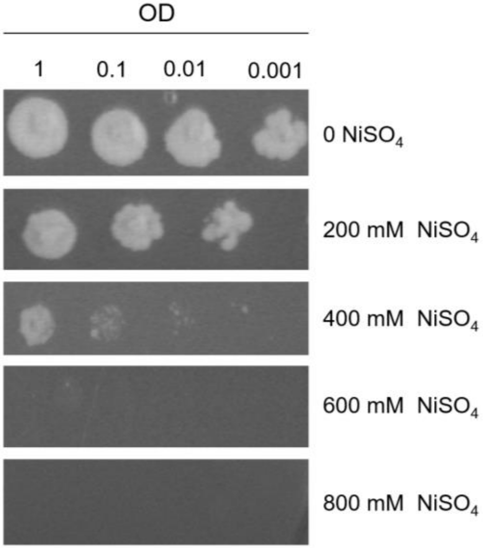

Figure S1: *Analysis of Ni tolerance and accumulation in yeast.* Ni minimum inhibitory concentration was determined in yeast cells transformed with the empty pADSL vector by spot assay. Yeast was grown overnight in 5 ml selective liquid YNB/G/W- at 28°C to early stationary phase. Yeast cells were then diluted to OD_{600} nm = 1, 0.1, 0.01 and 0.001 and spotted on YNB/G/W- plates supplemented with 0, 200, 400, 600, 800 µM $NiSO_4$. The concentration of 400 µM $NiSO_4$ reduced the growth of yeast transformed with empty plasmid at 1 and 0.1 OD, and inhibited the growth of yeast at 0.01 and 0.001 OD, whereas higher concentrations completely abolished yeast growth.
